# The interaction between island geomorphology and environmental parameters drives Adélie penguin breeding phenology on neighboring islands near Palmer Station, Antarctica

**DOI:** 10.1002/ece3.5481

**Published:** 2019-07-31

**Authors:** Megan A. Cimino, Donna L. Patterson‐Fraser, Sharon Stammerjohn, William R. Fraser

**Affiliations:** ^1^ Institute of Marine Sciences University of California, Santa Cruz Santa Cruz CA USA; ^2^ Polar Oceans Research Group Sheridan MT USA; ^3^ Institute of Arctic and Alpine Research University of Colorado Boulder CO USA

**Keywords:** Adélie penguin, breeding phenology, climate change, ecological threshold, environmental drivers, sea ice

## Abstract

Despite many studies on Adélie penguin breeding phenology, understanding the drivers of clutch initiation dates (CIDs, egg 1 lay date) is limited or lacks consensus. Here, we investigated Adélie penguin CIDs over 25 years (1991–2016) on two neighboring islands, Torgersen and Humble (<1 km apart), in a rapidly warming region near Palmer Station, Antarctica. We found that sea ice was the primary large‐scale driver of CIDs and precipitation was a secondary small‐scale driver that fine‐tunes CID to island‐specific nesting habitat geomorphology. In general, CIDs were earlier (later) when the spring sea ice retreat was earlier (later) and when the preceding annual ice season was shorter (longer). Island‐specific effects related to precipitation and island geomorphology caused greater snow accumulation and delayed CIDs by ~2 days on Torgersen compared to Humble Island. When CIDs on the islands were similar, conditions were mild with less snow across breeding sites. At Torgersen Island, the negative relationship between CID and breeding success highlights detrimental effects of delayed breeding and/or snow on penguin fitness. Past phenological studies reported a relationship between air temperature and CID, assumed to be related to precipitation, but we found air temperature was more highly correlated to sea ice, revealing a misinterpretation of temperature effects. Finally, contrasting trends in CIDs based on temporal shifts in regional sea ice patterns revealed trends toward earlier CIDs (4–6 day advance) from 1979 to 2009 as the annual ice season shortened, and later CIDs (7–10 day delay) from 2010 to 2016 as the annual ice season lengthened. Adélie penguins tracked environmental conditions with flexible breeding phenology, but their life history remains vulnerable to subpolar weather conditions that can delay CIDs and decrease breeding success, especially on landscapes where geomorphology facilitates snow accumulation.

## 1 INTRODUCTION

Breeding phenology in seabirds may be driven by internal and external cues, both biotic and abiotic, which theoretically aid in aligning food demand for chicks with peaks in food availability (Dawson, 2007; Perrins, 1970; Visser & Both, 2005). The ability of a species to synchronize their breeding chronology to optimal local conditions is thus considered crucial to population and breeding success, especially in polar regions where strong variability in seasonality, both natural and climate‐driven, can lead to short periods of favorable or unfavorable conditions, the latter possibly amplifying the potential for ecological mismatch (Visser & Both, 2005, Cushing, 1974, Schwartz, 2003; Thackeray et al., 2016). The recent and rapid warming at the poles in particular has inspired a number of studies to not only investigate the drivers of phenological trends on both global and regional scales, but also to understand the processes that affect the extent to which a species may be vulnerable to climate change (Black et al., 2018; Dunn & Winkler, 2010; Keogan et al., 2018; Parmesan & Yohe, 2003; Youngflesh et al., 2017). While many studies suggest the climate signal controlling spring breeding phenology is well understood (Walther et al., 2002; Wormworth & Sekercioglu, 2011), other studies report difficulties in teasing out the degree to which environmental factors drive phenology (Youngflesh et al., 2018), indicating further research is still necessary (e.g., identifying the correct time window or obtaining climate data at the appropriate scale).

Populations of Adélie penguins (*Pygoscelis adeliae*) along the Western Antarctic Peninsula (WAP) inhabit one of the most rapidly warming regions on Earth (Turner, Maksym, Phillips, Marshall, & Meredith, 2013; Vaughan et al., 2003), thus offering the opportunity to investigate the drivers of Adélie penguin breeding phenology in relation to a strongly trending climate change signature. The Adélie penguin is an ice‐dependent, migratory seabird with a circum‐Antarctic breeding distribution and a short temporal window in which to breed after returning to natal breeding colonies to rear chicks in the austral spring following migration from distant, generally ice‐covered wintering habitats (Fraser & Trivelpiece, 1996; Fraser, Trivelpiece, Ainley, & Trivelpiece, 1992). Being long‐distance migrants, Adélie penguins have little or no local information on breeding conditions at their natal colonies during winter, but it is likely that conditions at overwinter sites influence the timing of arrival at their breeding colonies by affecting the timing of migration (Ainley, 2002). Along the WAP, air temperatures have warmed, sea ice has declined and precipitation has increased (Obryk et al., 2016; Stammerjohn, Massom, Rind, & Martinson, 2012; Thomas et al., 2017), factors long‐known to negatively impact the demography and sustainability of Adélie penguin populations (Cimino, Fraser, Irwin, & Oliver, 2013; Cimino, Moline, Fraser, Patterson‐Fraser, & Oliver, 2016; Fraser & Patterson, 1997; Fraser, Patterson‐Fraser, Ribic, Schofield, & Ducklow, 2013; Fraser et al., 1992; Patterson, Easter‐Pilcher, & Fraser, 2003). In concert with these trends, evidence is accumulating to suggest that some spring‐associated events such as snowfall are changing in timing, magnitude, and in its persistence on the landscape (Kirchgäßner, 2011). These factors are episodic, but often produce devastating effects on breeding populations of Adélie penguins (Fountain et al., 2016; Massom et al., 2006).

In bird populations, the theoretical and empirical relationships between breeding phenology, resource availability and population dynamics has long been recognized (Lack, 1968), and with Adélie penguins these relationships have provided important insights into the probable mechanisms driving demography in the rapidly warming climate of the WAP related to changing precipitation patterns (Chapman, Hofmann, Patterson, Ribic, & Fraser, 2011; Cimino, Fraser, Patterson‐Fraser, Saba, & Oliver, 2014; Fraser & Patterson, 1997; Fraser et al., 2013; Patterson et al., 2003; Salihoglu, Fraser, & Hofmann, 2001). For example, it has been suggested that severe snowstorms and cold spring air temperature that prevent snowmelt can delay reproductive timing in Adélie penguins (Hinke, Polito, Reiss, Trivelpiece, & Trivelpiece, 2012; Lynch, Fagan, Naveen, Trivelpiece, & Trivelpiece, 2009; Lynch, Naveen, Trathan, & Fagan, 2012), and delayed breeding is often associated with lower breeding success (Youngflesh et al., 2018). However, Youngflesh et al. (2018) observed that despite many phenological studies on Adélie penguins (Barbraud & Weimerskirch, 2006; Emmerson, Pike, & Southwell, 2011; Hinke et al., 2012; Lynch et al., 2009, 2012; Smiley & Emmerson, 2016; Youngflesh et al., 2017), understanding what drives breeding phenology is still limited, and reported that high interannual phenological variability is normal and can occur in the absence of environmental variability.

Motivated by an interest in understanding how environmental factors influence phenology, we used 25 years (1991–2016) of Adélie penguin breeding phenology data to investigate and reveal the environmental drivers of clutch initiation date (CID), and the subsequent consequences to breeding success near Palmer Station, Anvers Island, WAP. We investigated the drivers of CID on two neighboring islands that are <1 km apart but differ significantly in landscape attributes that may affect breeding habitat quality. We hypothesized that ambiguity in previous findings could be related to the influence of island‐specific geomorphology on nest site microclimate (Fraser et al., 2013, Figure 1), which thus far has not been considered. In contrast to previous studies, our analyses also capitalized on local measurements of environmental parameters obtained as part of the Palmer Long‐term Ecological Research (LTER) program. This is noted because previous studies have typically used distantly recorded environmental conditions as proxies for local conditions; hence, we wanted to remove any ambiguities resulting from a possible scaling mismatch. Though our study location is only in one region of Antarctica, we aimed to provide useful insights for interpreting phenological dates as a proxy for species demographics or environmental forcing by considering both climate and landscape effects. Given the large climate signal on the WAP, we also reevaluated the absence of ecologically relevant phenological trends reported in past studies. We hypothesized that Adélie penguins track environmental conditions due to some flexibility in their breeding phenology, but remain vulnerable to subpolar weather conditions that can simultaneously delay breeding and influence breeding success.

**Figure 1:**
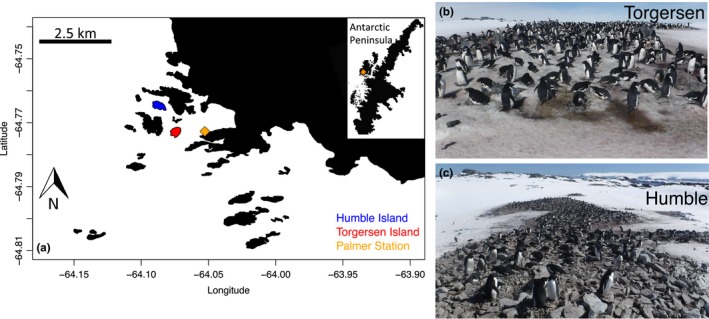
Palmer Station is located along the Western Antarctic Peninsula. (a) The study sites were located on Humble and Torgersen Island, near Palmer Station. (b) Torgersen Island often has more snow on the ground at the studied Adélie penguin colony than (c) Humble Island (photo credit Megan Roberts). Photos were taken on 16 November 2018

## 2 MATERIALS AND METHODS

### 2.1 Study site and penguin demographics

We studied Adélie penguin breeding phenology on Torgersen (TOR, 64°46′S, 64°5′W) and Humble (HUM, 64°46′S, 64°03′W) islands near Palmer Station, Antarctica (Figure 1). The TOR study colony is located on the north side of the island and essentially lies on a lower elevation gently sloped landscape, while the HUM study colony is located on the southeast side of the island on a slope at a slightly higher elevation than the TOR colony (Figure 1). Thus, HUM receives more wind scour even though both islands have equal exposure to the predominant northeasterly winds (Fraser & Patterson, 1997; Patterson et al., 2003).

Adélie penguins return annually to these rocky islands to begin nesting in mid‐October, with egg laying beginning in mid‐November. Adélie penguins typically lay two eggs (Ainley, 2002). In the vicinity of Palmer Station, the Adélie penguin population has declined by ~90% since the 1970s, with the subpopulation on TOR declining more steeply (8,119–1,200 breeding pairs; 85% decline) than on HUM (2,485–575 breeding pairs; 77% decline) during our 1991–2016 study period. Nest sites were monitored by noting the date of egg lay/loss, egg hatch/loss, and chick crèche each year. The crèche stage occurs when chicks (approx. 20 days of age) form groups independent of the nest (Ainley, 2002). The number of nests monitored annually ranged from ~50 to 200 with ~20 nests monitored toward the end of the time series due to population declines. The nest sites in the study were checked for the presence of adults, eggs and chicks every day (but see caveats below). Dated observations were recorded on a custom Julian calendar in which October 1 represented day 1, and the year represents the austral summer field season (e.g., 1991 includes October 1991 to March 1992). Some of the HUM data analyzed here were used in past phenological studies (Lynch et al., 2009, 2012; Youngflesh et al., 2017). All protocols were carried out in accordance with the approved guidelines of the Columbia University Institutional Animal Care and Use Committee (Assurance #AAAH8959).

Weather and sea ice conditions often prevented small boats from accessing the study sites. For this reason, lay and hatch dates were divided into two categories, true and estimated. For true dates, weather and ice did not impede daily checks, hence the exact timing of all nesting events were confirmed. However, if weather and ice induced gaps in daily checks, then lay and hatch dates at the next possible nest check were recorded as estimated because the exact dates were unknown. For all records with true dates, we calculated the mean and standard deviation in the lay date of egg 1 and egg 2, the days between these lay dates, the hatch date of chick 1 and chick 2, the days between these hatch dates, and the days between lay and hatch dates (incubation period). We determined whether there were significant differences between phenology at HUM and TOR using a linear mixed model (LMM) fit by maximum likelihood using lmer in the lme4 and lmerTest packages (Bates, Maechler, & Bolker, 2011) in the R statistical program (R Core Team, 2017) with the phenological parameter as the response variable, site as a fixed effect and year as a random effect (see Table 1). We also determined mean breeding success each year at each island by calculating the average of the number of chicks that crèched per breeding pair to compare with annual mean CIDs, or the day the first egg was laid.

**Table 1:** Mean and standard deviation of breeding phenology dates (days since September 30) at Humble (HUM) and Torgersen (TOR) Island from 1995 to 2016 when true dates were recorded

	HUM (*n* = 1707)	TOR (*n* = 2,838)	LMM results
Egg 1 lay day	43.11 ± 3.76 (*n* = 376)	45.89 ± 4.87 (*n* = 653)	1.83 ± 0.20 days *t* = 9.00, *p* < 2e−16
Egg 2 lay day	46.95 ± 4.09 (*n* = 439)	49.02 ± 4.11 (*n* = 766)	1.87 ± 0.18 days *t* = 10.65, *p* < 2e−16
Lay interval	3.12 ± 0.82 (*n* = 269)	3.09 ± 0.84 (*n* = 452)	−0.06 ± 0.06 days *t *= −0.9, *p* = .35
Chick 1 hatch day	79.58 ± 4.17 (*n* = 671)	81.18 ± 4.39 (*n* = 1,102)	1.54 ± 0.15 days *t* = 10.14, *p* < 2e−16
Chick 2 hatch day	80.47 ± 4.09 (*n* = 522)	81.86 ± 4.38 (*n* = 816)	1.61 ± 0.16 days *t* = 9.80, *p* < 2e−16
Hatch interval	1.15 ± 0.99 (*n* = 472)	1.16 ± 1.02 (*n* = 745)	1.6e−2 ± 5.9e−2 days *t* = 0.27, *p* = .79
Egg 1 incubation length	35.62 ± 1.58 (*n* = 283)	35.69 ± 1.65 (*n* = 447)	0.10 ± 0.12 days *t* = 0.82, *p* = .41
Egg 2 incubation length	33.43 ± 1.52 (*n* = 264)	33.62 ± 1.40 (*n* = 427)	0.22 ± 0.12 days *t* = 0.05, *p* = .05

Note

Incubation length is number of days between egg lay to egg hatch, lay interval is the number of days between egg 1 and egg 2 lay day, and hatch interval is the number of days between chick 1 and chick 2 hatch day. Linear mixed model (LMM) results show the phenology delays at TOR compared to HUM with year as a random effect (gray shading signifies significance at *p* < .05). The sample size (*n*) is in parentheses.

Because Adélie penguins have an incubation period of approximately 30–40 days (Ainley, 2002), there is a strong linear relationship between lay and hatch dates, which we used to adjust the estimated or unrecorded lay dates when weather or sea ice prevented colony access. We used all true lay and hatch dates to estimate the relationship (lay date ~ hatch date) using linear regression (*R* ^2^ = .88, Figure S1). Combining lay and hatch dates from both islands was appropriate because we found no significant difference between the incubation period at the two islands across years (Table 1, LMM *t*‐statistic = 0.82, *p* = .41). For this linear regression between lay and hatch dates, 91% of the predicted lay dates were within ±2.5 days of the true lay dates, 83% were within ±2 days and 70% were within ±1.5 days. An error range of ~2 days may seem considerable but it should be noted that a 1.5‐day interval was within our measurement resolution (e.g., if the colony was visited on Day 1 in the morning and on Day 2 in the evening). Therefore, we used this linear regression model to adjust estimated lay dates and dates where the true/estimated identifier was not recorded. The latter was especially true for dates prior to 1995, or before protocols were refined to accommodate the environmental conditions that might affect island access and thus how date‐specific data were interpreted. Thus, prior to 1995, we used the hatch dates to check the lay dates, and if the predicted lay date was within ±2.5 days of the recorded lay date, the record was retained (95% of the data) and all others were removed from further analysis. Similarly, after 1995, if both lay and hatch dates were estimated, we only retained records if the predicted and recorded lay dates were within ±2.5 days (57% of the data).

We computed mean CIDs for each island for each year. We removed 2013 (for HUM and TOR) and 2005 (for HUM) from further analysis due to low sample sizes resulting from unusually poor weather or sea ice conditions. For both years, <35% of the data had a true hatch or lay date, and the number of trues was <5. For all other years, we compared adjusted and unadjusted mean CIDs by island using Pearson's correlation to determine the strength of the correlation. We tested whether the CIDs differed between sites and years using a LMM as described above with CID as the response variable, site as fixed effect and year as a random effect. Further, to identify which years had significantly different CIDs between sites, we used a separate Welch two sample *t* test with the Benjamini–Hochberg false discovery rate used as a correction for multiple testing (Benjamini & Hochberg, 1995; Pike, 2011) (deemed significant at *p* < .05) for each year to test for differences between island means using all CID records within that year. Additionally, for each island, we calculated the anomaly in the mean CIDs by subtracting the time series CID mean from each season's mean CID.

Adélie penguin chick fledging mass was measured at HUM as chicks gathered on beaches (for methods and drivers see Chapman et al., 2011; Cimino et al., 2014; Salihoglu et al., 2001). Because Cimino et al. (2014) did not include phenology as a candidate driver of chick mass, we used linear regression to determine whether there was a significant linear relationship between annual mean chick mass and CID on HUM. We also used linear regression to determine whether there was a relationship between annual mean CID and breeding success on HUM and TOR.

### 2.2 Environmental predictors of clutch initiation dates

We aimed to understand if CID was related to environmental drivers. Adélie penguins generally return to their colonies in October for courtship and nest building prior to laying eggs in November. Therefore, conditions in the late austral winter and early spring may influence CIDs. Other studies have shown that CID is related to October air temperature, hypothesized to be indicative of snow on the ground (Hinke et al., 2012; Lynch et al., 2009, 2012). It was thought that warm October temperature facilitates snowmelt, making for suitable nesting areas where penguins can build nests. Therefore, we used mean October air temperature and November snow depth as predictors of CIDs, both measured locally at Palmer Station and obtained from the Palmer LTER data archives (http://pal.lternet.edu/data/). We also calculated the number of days with precipitation (rain or snow >0 cm) in October to compare with observations at nearby Faraday/Vernadsky Station (discussed below).

We obtained sea ice parameters (sea ice retreat day, concentration and area in October and November), because these may influence access to the colony, access to open water from the colony, be related to wintering locations, parental condition, and/or migration distance/timing of arrival. Sea ice retreat day within 200 km of Anvers Island (Stammerjohn, Martinson, Smith, & Iannuzzi, 2008) was defined as the day in which sea ice concentration fell below 15%. Monthly sea ice area was defined as the area covered by sea ice with concentrations >15% within the Palmer LTER study grid. Monthly sea ice concentration within 200 km of Anvers Island was also used. Sea ice records used here from 1979 to 2016 are available on the Palmer LTER data archive.

Climate indices may be related to CID through teleconnections due to both sea ice and weather being linked to the El Niño Southern Oscillation (ENSO) (Renwick, 2002; Stammerjohn, Martinson, Smith, & Yuan, 2008; Steinberg et al., 2015). We obtained El Niño 3.4 (Nino3.4) information from the NOAA National Weather Service, National Centers for Environmental Prediction, Climate Prediction Center (www.cpc.ncep.noaa.gov/). Nino3.4 reflects warm El Niño (high Nino3.4) and cold La Niña (low Nino3.4) conditions in the equatorial Pacific (Carleton, 2003; Kwok & Comiso, 2002). We also used the Multivariate ENSO Index (MEI), which reflects both the ocean–atmosphere system and was obtained from the NOAA Earth System Research Laboratory, Physical Sciences Division (https://www.esrl.noaa.gov/psd/enso/mei/index.html).

When the breeding/phenology monitoring sites on HUM and TOR were established in late October or early November from 1998 to 2016, snow depth was usually measured at the colony edge near the groups of monitored nests. This measurement was taken before the mean CID and was an indication of snow depth at the sites before clutch initiation. We used these data to investigate the difference in snow depth between the two locations, which was likely related to the influence of island‐specific geomorphology on snow accumulation (Figure 1; Fraser et al., 2013). We calculated the mean and standard deviation in snow depth by island and year and then calculated the difference between the mean snow depth on HUM and TOR as an indication of island‐specific effects. A LMM with snow depth as the response, site as a fixed effect and year as a random effect was used to test whether snow depth differed between sites and years.

Finally, we obtained 1979–2015 precipitation information for Faraday/Vernadsky (64°15′S, 64°16′W) acquired by the British Antarctic Survey (https://legacy.bas.ac.uk/cgi-bin/metdb-form-1.pl?table_prefix=U_WMC,U_MET&acct=cmet). Precipitation information was recorded as part of routine synoptic observations under “Present weather,” which refers to weather at the time of observation. Observations every 3 hr were recorded as numerical codes (WMO, 1995). The actual amount of precipitation was not recorded. To match and extend our Palmer Station precipitation record, we computed the number of precipitation days (solid precipitation in the form of snowflakes or grains) in October from 1979 to 2015. The amount of snow on the ground in November is related to how much and often it snowed in October (similar to Kirchgäßner, 2011). Our analyses suggested that rain/snow at Palmer Station equated to snow at Faraday/Vernadsky, likely due to Faraday/Vernadsky being >50 km south of Palmer Station.

### 2.3 Statistical analyses

**Figure 2:**
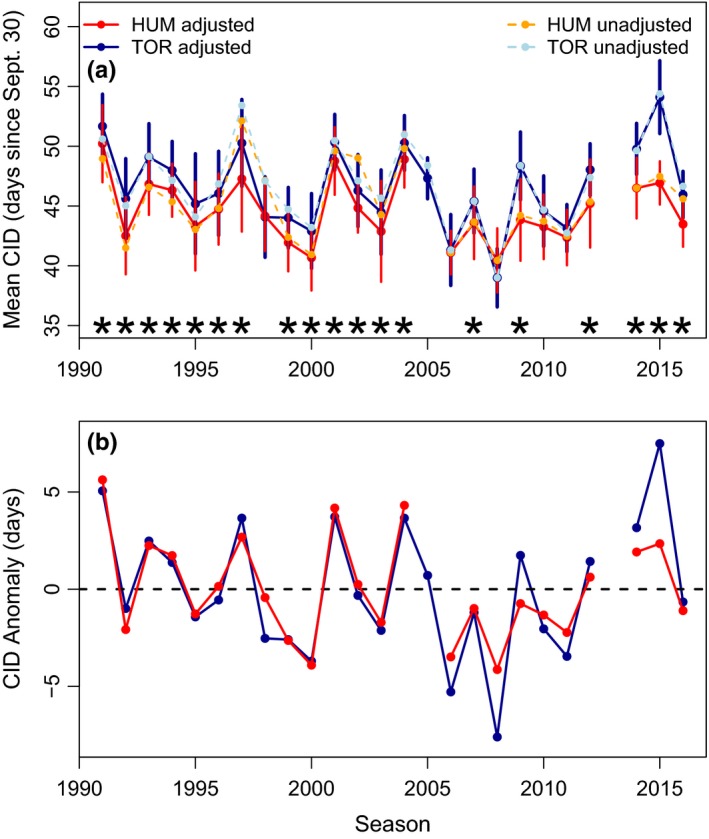
Variation in clutch initiation dates (CID) over time. (a) Mean CID for adjusted and unadjusted dates at Humble (HUM) and Torgersen Island (TOR). Stars represent significant differences between adjusted CID at HUM and TOR for each year. Vertical lines are the standard deviation for adjusted CIDs. (b) Anomalies in mean CID for adjusted mean CIDs by island. Positive anomalies are CIDs occurring later than average while negative anomalies occur earlier than average. For TOR, 2013 was removed and for HUM, 2005 and 2013 were removed due to low sample size

We hypothesized that in years without significant differences in mean CIDs (no stars in Figure 2a) between the two islands, the environmental conditions would be more benign (e.g., intermediate sea ice/air temperature and low snow) and more similar to each other than during years when CID differed between islands. Thus, we grouped environmental parameters into two categories by year, (a) significant difference in CID or, (b) nonsignificant difference in CID. We did a *t* test between the environmental conditions in significant versus nonsignificant years to determine whether there was an environmental difference. We tested all environmental variables measured at Palmer Station described above. Similarly, we tested for differences in breeding success and chick mass during significant versus nonsignificant years using a *t* test. We also calculated the difference in days between mean CID at HUM and TOR each year and used linear regression to determine whether this difference was related to the environment (Difference in CID ~ environmental variable). A separate linear regression was run for each environmental variable.

Next, we tested whether interannual variability in mean CIDs co‐varied with environmental variables from 1991 to 2016. We used generalized additive models (GAMs) using the “mgcv” package in R. The smoothness parameter (s) was estimated using generalized cross‐validation (Wood, 2006). GAMs are flexible and capable of fitting complex nonlinear relationships. Prior to GAM fitting, we investigated the relationship between predictor variables by calculating the Pearson correlation coefficients (Figure S2) because collinear variables could lead to model overfitting and did not include any variables in the same model when the correlation was above .55. For model selection, the Akaike information criterion for small sample size (AIC_c_) identified the models that accounted for the most variation, selected the model with the best balance between bias and precision, and avoided over fitting (Burnham & Anderson, 2002). We considered models with a ΔAIC_c_ < 2 to have substantial support and models with ΔAIC_c_ > 10 to have no support. We also reported the percentage of deviance explained, the Adjusted *R* ^2^ and Akaike weight as indicators of model performance. We ran three suites of models using, (a) mean annual CID at TOR, (b) mean annual CID at HUM, and (c) an average of mean CIDs at HUM and TOR as response variables, with a combination of predictor variables. We report all models with ΔAIC_c_ < 10. For each GAM, the effect of each environmental predictor was plotted to visually inspect the functional form to confirm the same relationship was seen within and across the three model suites. This general approach has been used in many studies to look at the environmental drivers of seabird demographics (e.g., Bond et al., 2011; Cimino et al., 2014; Dehnhard, Ludynia, Poisbleau, Demongin, & Quillfeldt, 2013; Santora, Veit, Reiss, Schroeder, & Mangel, 2017).

As studies often report no trends in seabird phenology over short time series (Keogan et al., 2018), we used Faraday/Vernadsky precipitation and sea ice records to extend our time series to 1979 to estimate the possible response in CID. We refitted our two best performing GAMs at TOR by replacing November snow depth with the number of precipitation days at Faraday/Vernadsky from 1991 to 2015 (years with available CID and Faraday/Vernadsky data). We then applied this model onto the full time series of Faraday/Vernadsky precipitation days and sea ice data from 1979 to 2015 to estimate CID. We tested for a trend in the estimated Adélie penguin mean CID over time. It has been recently recognized that sea ice trends began leveling off or reversing along the WAP since about 2008–2009 when a local minimum in annual ice season duration was observed, after which there was an increase in the length of the annual ice season (Henley et al., 2019; Schofield et al., 2017), at least until ~2016. Therefore, using linear regression, we tested for trends in observed CIDs and predicted CIDs over time by assessing the time series before and after this local minimum. For the GAM that included sea ice retreat day, we omitted 1989 from the linear regression analysis because 1989 was an extreme outlier and predicted an earlier CID by nearly a month. Such an extreme shift in CID is unlikely, especially given other factors like photoperiod or hormones acting as other cues (Dunn & Winkler, 2010).

## 3 RESULTS

### 3.1 Reproductive phenology

On average, egg 1 was laid 1.8 days later on TOR than HUM, or November 12 (day 43) and November 15 (day 46) for HUM and TOR (Table 1). Egg 2 was laid approximately 3 days later on both islands, on November 16 and 18 for HUM and TOR, which was 1.9 days later on TOR (Table 1). Mean hatch day for chick 1 was ~36 days after egg 1 was laid, on December 19 and 20 for HUM and TOR, which was 1.5 days later on TOR. Mean hatch day for chick 2 was approximately one day after chick 1 hatched, following an incubation period of ~33 days on both islands. On average, chick 2 hatched on December 19 and 21 for HUM and TOR, which was 1.6 days later for TOR (Table 1). There were no significant or biologically meaningful differences between HUM and TOR in the number of days between egg 1 and 2 lay date, days between egg 1 and 2 hatch date, or incubation length for egg 1 and 2.

In general, the annual mean adjusted and unadjusted CIDs at HUM and TOR were similar (Pearson's *R*, HUM *R* = .88, *p* = 1.5e‐08; TOR *R* = .96, *p* = 7.1e‐14) and had the same variability over time (Figure 2a). The adjusted mean CIDs were often statistically different between islands and years (*t* test, *p* < .05; in Figure 2a the stars indicate significance by year). A LMM showed that across all years TOR penguins bred later than HUM penguins by an average of 1.99 ± 0.32 days (*t*‐statistic = 6.13, *p* = 2.3e‐6). The overall mean lay date for all adjusted mean CIDs was November 14 and 16 for HUM and TOR. Anomalies across years in mean CIDs ranged from approximately ±5.6 and ±7.6 days for HUM and TOR (Figure 2b).

### 3.2 Environmental conditions versus clutch initiation date

**Figure 3:**
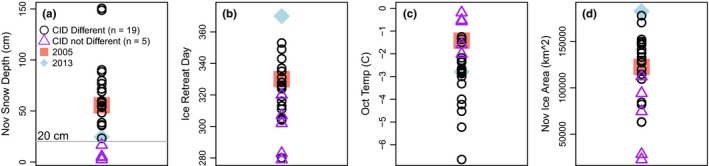
Environmental parameters grouped by years that had significant and nonsignificant differences in clutch initiation date (CID) between Humble and Torgersen Island (Figure 2a). (a) November snow depth, (b) ice retreat day, (c) October air temperature, and (d) November ice area between years with different and not different CID. The two years that were excluded from the analysis (2005 and 2013) are shown for reference

There were 19 years where CIDs were significantly different between islands (Figure 2a) and only five years where CIDs were not different, which we acknowledge is a small sample size. During years when HUM and TOR CIDs were not significantly different, November snow depth as measured at nearby Palmer Station was significantly less (Figure 3a), ice retreat day was significantly earlier (Figure 3b), October air temperature was significantly warmer (Figure 3c) and November ice area was significantly lower (Figure 3d) than in years when CIDs were significantly different between islands (*t* test, *p* < .05). While there was overlap in the values of most environmental parameters between significant and nonsignificant years (Figure 3b–d), there was no overlap in snow depth, with clearly separated groups at ~20 cm (Figure 3a). Similarly, linear regression results with the difference in mean CID at TOR and HUM as the response against each environmental predictor showed a significant positive relationship with snow depth (*y* = 0.03*x* + 0.49, *R* ^2^ = .41, *p* = .0007), and a significant negative relationship with ice retreat day (*y* = 0.04*x* − 10.42, *R* ^2^ = .22, *p* = .02), November ice area (*y* = 2.02*x *− 0.31, *R* ^2^ = .27, *p* = .01), and El Niño indices (October Nino3.4, *y* = 0.74–17.90, *R* ^2^ = .25, *p* = .01; November Nino3.4, *y* = 0.67–15.87, *R* ^2^ = .26, *p* = .01; October‐November MEI, *y* = 0.76 + 1.77, *R* ^2^ = .26, *p* = .01). Interestingly, there was no significant relationship between the difference in mean CID and air temperature (*y* = −0.23*x* + 0.22, *R* ^2^ = .05, *p* = .31).

**Figure 4:**
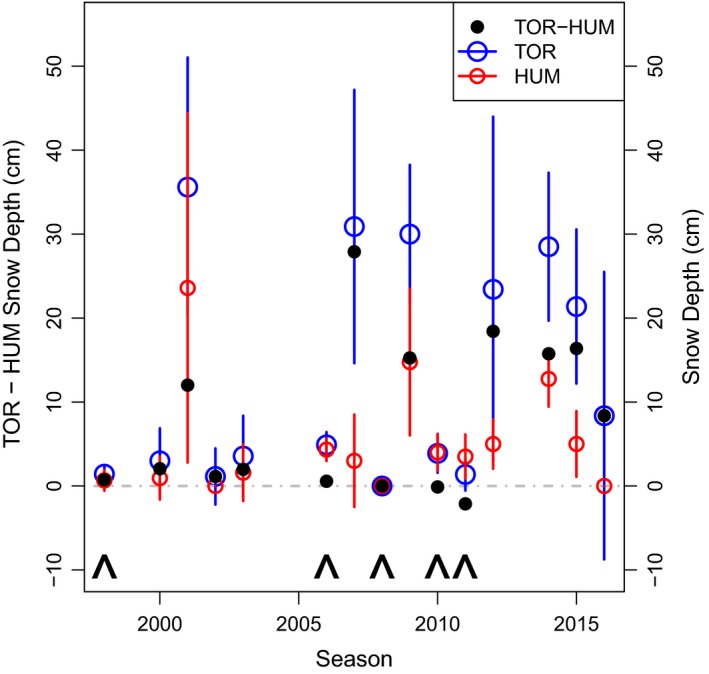
Snow depth at Humble (HUM) and Torgersen (TOR) Island and the difference between mean snow depths at the two sites for each season. The mean and standard deviation in TOR (blue) and HUM (red) snow depth measured on the same day within each season. The difference between mean snow depth measured on TOR and HUM (black) for each season. Positive values indicate higher snow depth on TOR. The gray dashed lines indicates zero. The arrows (^) indicate years with nonsignificant differences in mean CID

Because mean CIDs were related to snow depth, we further investigated island‐specific conditions by comparing snow depth measured when nest monitoring sites were established from 1998 to 2016. Mean snow depth measurements at TOR and HUM were correlated (*R* = .78, *p* = .0006), but there were years of noticeably more snow on TOR (Figure 4). In many years, mean snow depth at each island was <5 cm at the time of the measurement and in those years, the difference in snow depth between islands was small. However, there were also years with mean snow depth > ~10 cm and in those years, the difference in mean snow depth between islands was often greater (Figure 4). LMM results revealed snow depth was significantly deeper by 6.32 ± 1.09 cm on TOR than HUM (*t*‐statistic = 5.79, *p* = 1.7e‐08). In the five years when the mean CID was not significantly different between islands (arrows in Figure 4), these were the lowest absolute differences in snow depth between islands (<0.75 cm in 1998, 2006, 2008, 2010) and/or were the only cases of HUM having slightly more snow (0.11 and 2.12 cm more in 2010 and 2011).

GAMs were used to relate HUM, TOR and an average of HUM and TOR CIDs to environmental parameters. Our best models (ΔAIC_c_ < 2) performed well and explained most of the variance for HUM (*R* ^2^ = ~.6), TOR (*R* ^2^ = ~.77) and the average of HUM and TOR CIDs (*R* ^2^ = ~.7) (Table 2). The best models used only two predictor variables. The models with the highest Akaike weight included snow depth and air temperature for HUM (58%), snow depth and ice retreat day for TOR (49%), and snow depth and ice retreat day for the average of HUM and TOR (43%). The top three models (models 1, 2, and 7) performed better than all other models tested (ΔAIC_c_ < 2, Table 2). The parameters tested were also somewhat related to each other (Figure S2). For example, Nino3.4 and MEI were related to air temperature (*R* = .63, .66) and sea ice conditions (*R* = .6–.7); air temperature was generally negatively related to sea ice variables (*R* = .5–.8). In contrast, snow depth was not strongly related to any environmental variables (*R* < .53, Figure S2).

**Table 2:** General Additive Models relating mean clutch initiation date (CID) at Humble and Torgersen Island to snow, sea ice and climate variables from 1991 to 2016

	Model	*R* ^2^	Dev.Explained (%)	AIC_c_	ΔAIC_c_	Weight
Torgersen Island CID ~						
**2**	**Nov_SnowDepth + s(IceRetreatDay)**	**.76**	**78.41**	**103.8**	**0**	**0.49**
**1**	**Nov_SnowDepth + s(Nov_IceArea)**	**.77**	**79.54**	**104.3**	**0.5**	**0.38**
8	Nov_SnowDepth + s(Oct_IceArea)	.73	76.05	108.6	4.8	0.05
7	Nov_SnowDepth + Oct_Temp	.7	72.24	109.6	5.8	0.03
10	Nov_SnowDepth + Nov_Nino3.4	.6	71.85	110.0	6.2	0.02
6	Nov_SnowDepth + s(OctNov_MEI)	.72	75.96	111.2	7.4	0.01
3	Nov_SnowDepth + Oct_SIC	.68	70.30	111.3	7.5	0.01
5	Nov_SnowDepth + s(Oct_Nino3.4)	.7	74.72	112.0	8.2	0.01
Humble Island CID ~						
**7**	**Nov_SnowDepth + Oct_Temp**	**.59**	**62.95**	**100.1**	**0**	**0.58**
2	Nov_SnowDepth + s(IceRetreatDay)	.56	60.93	102.8	2.7	0.15
3	Nov_SnowDepth + Oct_SIC	.53	57.54	103.4	3.3	0.11
1	Nov_SnowDepth + s(Nov_IceArea)	.52	57.40	105.4	5.3	0.04
13	s(IceRetreatDay)	.44	48.27	107	6.9	0.02
16	s(Oct_Temp)	.43	46.62	107	6.9	0.02
8	Nov_SnowDepth + s(Oct_IceArea)	.52	59.87	107.4	7.3	0.02
6	Nov_SnowDepth + s(OctNov_MEI)	.45	49.64	107.5	7.4	0.01
9	Nov_SnowDepth + Nov_SIC	.44	49.06	107.7	7.6	0.01
4	Nov_SnowDepth	.4	42.66	107.8	7.7	0.01
10	Nov_SnowDepth + Nov_Nino3.4	0.44	48.82	107.9	7.8	0.01
5	Nov_SnowDepth + s(Oct_Nino3.4)	.48	56.28	109.4	9.3	0.01
Mean Torgersen & Humble Island CID ~						
**2**	**Nov_SnowDepth + s(IceRetreatDay)**	**.71**	**74.22**	**98.6**	**0**	**0.43**
**7**	**Nov_SnowDepth + Oct_Temp**	**.69**	**71.55**	**99.7**	**1.1**	**0.25**
**1**	**Nov_SnowDepth + s(Nov_IceArea)**	**.7**	**73.67**	**100.4**	**1.8**	**0.18**
3	Nov_SnowDepth + Oct_SIC	.64	67.55	102.8	4.2	0.05
8	Nov_SnowDepth + s(Oct_IceArea)	.67	72.17	103.6	5.0	0.03
10	Nov_SnowDepth + Nov_Nino3.4	.62	65.00	104.6	6.1	0.02
6	Nov_SnowDepth + s(OctNov_MEI)	.65	71.30	105.9	7.3	0.01
5	Nov_SnowDepth + s(Oct_Nino3.4)	.64	69.97	106.5	7.9	0.01
4	Nov_SnowDepth	.55	56.48	107.1	8.5	0.01
9	Nov_SnowDepth + Nov_SIC	.55	59.35	108.3	9.6	0

Note

The models are described by the adjusted *R* ^2^, AIC_c_ for small sample size, ΔAIC_c_ (difference from the lowest AIC_c_; amount of information lost), and Akaike weight showing relative model support or probabilities. Models are sorted by ascending ΔAIC_c_, models with substantial support (ΔAIC_c_ < 2) are in bold and only models with a ΔAIC_c_ < 10 are shown. Model variables: Nov_SnowDepth is average depth of snow at a snow stake in November; Nov and Oct_IceArea is the total area of sea ice occupying the WAP LTER grid in each month; Nov and Oct_SIC is the mean sea ice concentration around Anvers Island that month; IceRetreatDay is the day of sea ice retreat within 200 km of Anvers Island; Oct_Temp is average air temperature in Oct; OctNov_MEI is average MEI during Oct and Nov; Nov and Oct_Nino3.4 is monthly averaged Nino3.4 conditions.

**Figure 5:**
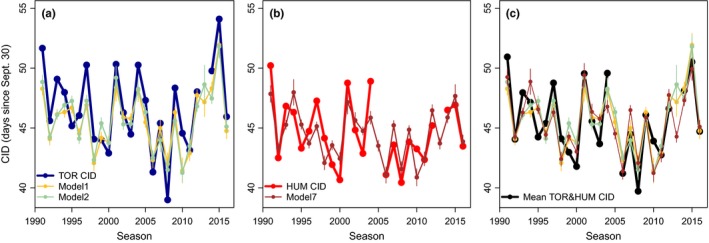
Observed mean clutch initiation date (CID) compared to modeled CID for (a) Torgersen (TOR) only, (b) Humble (HUM) only, and (c) the mean of HUM and TOR. Vertical bars are standard error estimates. Only models with substantial support were plotted (ΔAIC_c_ < 2)

**Figure 6:**
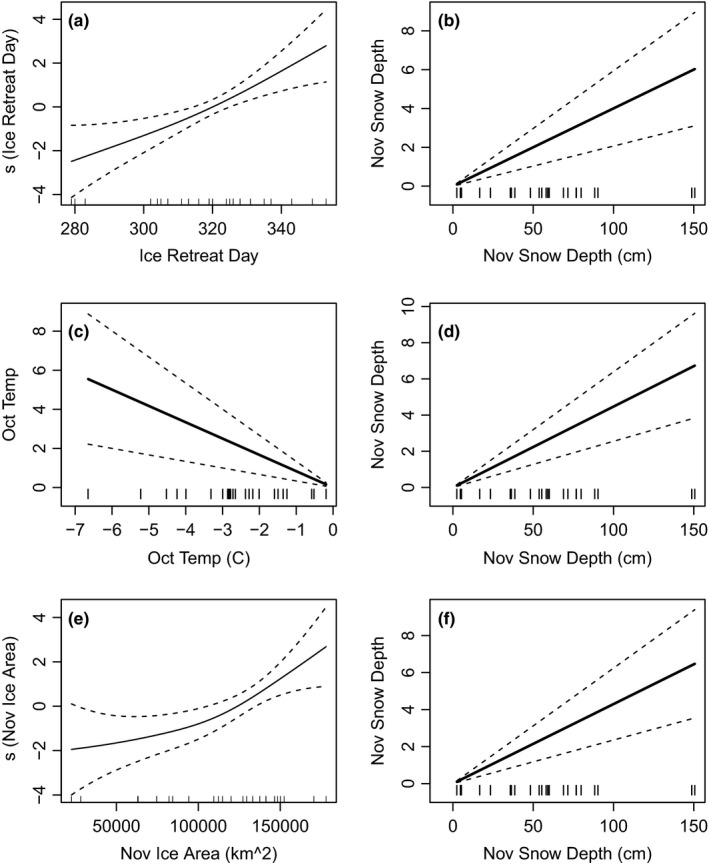
Fitted generalized additive model results for the mean of Humble and Torgersen Island CID as a function of (a) sea ice retreat day and (b) November snow depth from model 2; (c) October temperature and (d) November snow depth from model 7; and (e) November sea ice area and (f) November snow depth from model 1. The effect on CID is represented as a spline (s) of ice retreat day and November ice area. The dotted line is the 95% confidence interval

The best performing models were in good agreement with the data, being on average within ±1.4 days of observed CIDs across all years (Figure 5). There were four unique terms in models 1, 2, and 7, including November snow depth (included in all models), ice retreat day, October temperature, and November ice area (Table 1). Snow depth and air temperature were linearly related to CID while ice retreat day and ice area had a slight nonlinear relationship with CIDs (Figure 6). Overall, CIDs were later when snow depth was deeper, ice retreat day was later, air temperature was cooler and ice area was greater (Figure 6). The fitted relationships were similar when visualizing predictor variables for only HUM or TOR CID models.

### 3.3 Clutch initiation date versus breeding success

**Figure 7:**
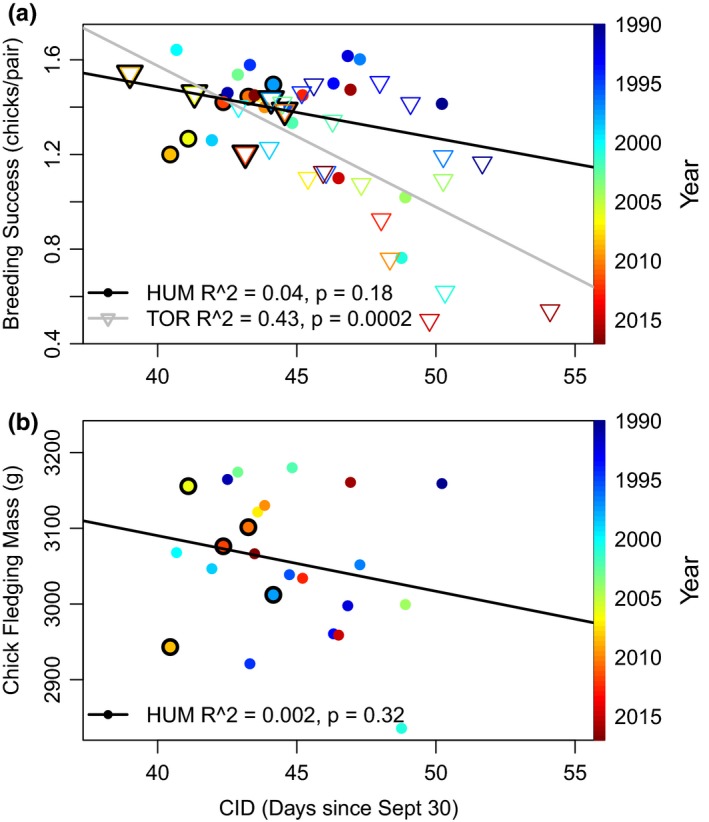
Mean clutch initiation date (CID) compared to (a) mean breeding success at Humble (HUM) and Torgersen (TOR) Island and (b) mean chick fledging mass at HUM. The circles and triangles that are outlined in black correspond to the five years when CIDs were not significantly different between islands

At both TOR and HUM, a negative linear relationship was revealed between CID and breeding success, but it was only statistically significant at TOR (*p* < .05, Figure 7a). During the five years, when CID did not differ between HUM and TOR (Figure 2a), mean breeding success was significantly higher (*t* test, *p* = .02) than during other years at TOR but not significantly different at HUM (*p* = .7). At both islands, however, breeding success was relatively high (>1.2 chicks/pair) in those five years (Figure 7a). Finally, there was no significant relationship between CID and chick fledging mass at HUM nor was chick mass statistically different during the five years when CIDs were not different between islands (Figure 7b).

Several years in the time series had unusual environmental conditions that were further considered in relation to breeding success. The years 2001 and 2015 had the deepest snow accumulation as measured at Palmer Station (Figure 3) and 2015 was also a high sea ice year (area > 170,000 km^2^, retreat day of 349; Figure 3, Figure S3a). At TOR, the years with the lowest mean breeding success were 2001 (0.61 chicks/pairs), 2014 (0.5 chicks/pair), and 2015 (0.54 chicks/pair), which were three of the five years with a mean CID ≥ day 50 (Figure 7a). At HUM, there were no years with mean breeding success <0.75 or CID >day 49.

### 3.4 Trends in clutch initiation date

**Figure 8:**
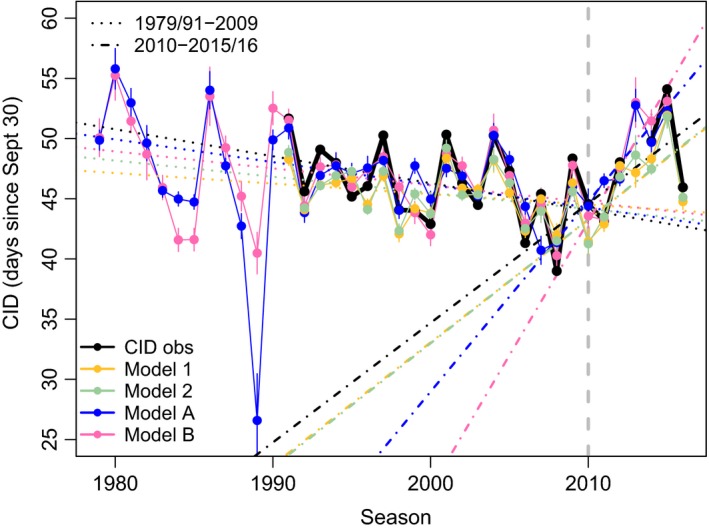
Time series of mean clutch initiation date (CID) observations (obs) and model predictions for Torgersen Island (see Tables 2 and 3). Vertical bars are standard error estimates. Sea ice began rebuilding in 2010 (gray dashed line). Linear regression lines for each time series of data for the two time periods are shown, before and after 2010. For Model A/B, precipitation data were not available for 2016

For TOR, we tested for a trend in CID over time by extending the precipitation time series to 1979 (Figure 3b). Models A and B included the number of precipitation days and November ice area, and precipitation days and ice retreat day, respectively (Table 3, Figure 8). As with previous models (Table 2), models A and B performed well and explained a high proportion of the variance (Table 3). CIDs were later when there were more precipitation days, ice retreated later, and ice area was higher (as in Figure 6). We ran linear regressions to test for a trend in expected CID over time using two sets of years, 1979/1991–2009 and 2010–2015/2016 (Figure 8). From 1979/1991 to 2009, a negative trend was present in expected CIDs over time, which was significant or marginally significant for model A (−0.19 ± 0.06 days/year, *p* = .005), model B (−0.14 ± 0.07 days/year, *p* = .07), and CID observations (−0.23 ± 0.13 days/year, *p* = .11). From 2010 to 2015/16, a positive trend was present in expected CID over time, which was significant or marginally significant for model A (1.57 ± 0.46 days/year, *p* = .03), model B (2.22 ± 0.48 days/year, *p* = .01), and model 2 (1.0 ± 0.55 days/year, *p* = .12). The slope describing the positive trend for CID observations from 2010 to 2016 was 0.99 ± 0.67 days/year (*p* = .2). While the modeled and observed CID did not always have statistically significant trends, the trend lines across all time series were very similar and in the same direction (Figure 8). Overall, the trends revealed a ~4–6 day advance in CID from 1979/1991 to 2009, and ~7–10 day delay in CID from 2010 to 2015/2016.

**Table 3:** General Additive Models relating mean clutch initiation date (CID) at Torgersen Island to Faraday/Vernadsky precipitation days and sea ice variables from 1991 to 2015

Model		*R* ^2^	Dev.Explained (%)	AIC_c_	ΔAIC_c_	Weight
Torgersen Island CID ~						
A	s(Precip_days) + IceRetreatDay	.59	63.00	114.7	0	0.87
B	s(Precip_days) + s(Nov_IceArea)	.82	89.50	118.5	3.8	0.13

Note

See Table 3 for information on parameters reported in the table. Model variables: Precip_days is the number of days with snow precipitation at Faraday/Vernadsky; IceRetreatDay is the day of sea ice retreat within 200 km of Anvers Island; Nov_IceArea is the total area of sea ice occupying the WAP LTER grid in November.

## 4 DISCUSSION

We believe this is the first study to report differences in phenology and breeding success between colonies on closely neighboring islands, highlighting the importance of small‐scale island‐specific landscape attributes in a region where storm and precipitation events are becoming more frequent. In the following discussion sections, we consider the relative roles of sea ice and precipitation as primary, large‐scale (Section 4.1) and secondary, smaller‐scale drivers (Section 4.2), respectively, including the role of the latter especially as a source of delayed breeding on TOR due to interactions with a landscape that is more prone to snow accumulation. In this context, we discuss why air temperature can be indicative of CID but not a main driver (Section 4.3) and explore why environmental triggers are critical to Adélie penguins (Section 4.4). In the last two sections, we discuss the negative association between delayed CIDs and breeding success (Section 4.5) and the ecological/evolutionary relevance of trends in Adélie penguin phenology (Section 4.6).

### 4.1 Sea ice as the primary driver of clutch initiation date

Sea ice is a key driver of phenology across many trophic levels (Barbraud & Weimerskirch, 2006; Cherry, Derocher, Thiemann, & Lunn, 2013; Ramírez et al., 2017; Saba et al., 2014). We found that CID models that included sea ice and precipitation described most of the variation (~60%–80%) in CIDs, which accords with the early observation that annual sea ice variability is a major driver of Adélie penguin life history patterns (cf. Fraser et al., 1992). As with other seabirds, the initial commencement of the breeding season in Adélie penguins is likely hormonal, induced by a lengthening photoperiod in spring that leads to changes in behavior including migration to natal nesting colonies (Ainley, 2002; Murton & Westwood, 1977; Van Tienhoven, 1961). However, photoperiod alone cannot explain the interannual variability in CIDs we observed because it does not change from year‐to‐year in comparison with environmental variables such as sea ice, which can have both direct and indirect effects on breeding chronology. During some years, for example, sea ice can physically impede penguins from completing their migration back to natal colonies (e.g., Ainley, 2002; Ainley, LeResche, & Sladen, 1983), thus forcing a delay until the ice cover diminishes to some optimal state (the habitat optimum hypothesis, Fraser & Trivelpiece, 1996). Austral winter/spring sea ice conditions may also influence parental body condition, and in turn affect CIDs via feedback mechanisms that delay breeding in response to food web variability. The presence or absence of sea ice influences the magnitude of phytoplankton blooms and krill abundance, which may impact prey availability at the earliest stages of the breeding cycle (Atkinson et al., 2008; Saba et al., 2014; Steinberg et al., 2015).

Adélie penguins evolved in a polar climate, and their life history strategies are tied to sea ice cycles that regulate key aspects of their foraging ecology and demography, including survival and reproduction (Cimino et al., 2014; Forcada, Trathan, & Murphy, 2008; Fraser & Hofmann, 2003; Fraser & Trivelpiece, 1996; Fraser et al., 1992). Considering such a life history from an evolutionary perspective, it is not surprising that our results suggested that sea ice dynamics were a primary driver of CID variability, even though, admittedly, challenges remain insofar as identifying the exact mechanisms responsible for this association.

### 4.2 Precipitation as a secondary driver of clutch initiation date

Precipitation was the other significant variable determining CIDs. However, because Adélie penguins have no local knowledge about snow conditions at their breeding colony when they initiate their return migration, and snow conditions can be both unpredictable and highly variable annually, it is equally unsurprising that precipitation plays a secondary role as a determinant of CID variability. While snow caused delayed breeding on both islands, the effect of snow was magnified on TOR likely due to the influence of island geomorphology on snow deposition (Fraser & Patterson, 1997; Patterson et al., 2003). This island‐specific snow accumulation mechanism that caused differences in CIDs on the two islands is supported by long‐term studies where island geomorphology and, thus, snow deposition and accumulation, results in differing availabilities of suboptimal habitat on each island. The island in the Palmer Station study region with the greatest amount of suboptimal habitat is Litchfield Island, where the Adélie penguin population went extinct in 2007 (Fraser et al., 2013) after a prior occupation of ~500 years (Emslie, Fraser, Smith, & Walker, 1998), emphasizing the Adélie penguin life history is no longer suited to the emerging warm and moist WAP ecosystem.

There also appeared to be a threshold effect between snow depth and CIDs, illustrated by November snow depths >20 cm corresponding to delayed breeding on TOR (Figure 3). In many of those years, the difference in snow depth between the two islands was ~10–25 cm and on average, TOR snow depth was ~6 cm deeper than HUM, which further supports a threshold effect. Biologically, it makes sense that there is a threshold snow depth that delays CIDs, as from an evolutionary perspective birds must respond to thresholds that may affect fitness.

We acknowledge that snow depth in the models was measured at Palmer Station; therefore, the differences between observed and modeled CID as well as model performance at the two islands could be due to the islands having different landscapes than Palmer Station, and Palmer Station snow depth not being fully representative of island conditions. The islands likely receive greater exposure to wind scour than Palmer Station, but Palmer Station snow data does capture the overall regional snow conditions and anomalies each year.

### 4.3 Air temperature as an indicator of clutch initiation date

Many phenophases of plants and animals (including penguins) correlate with spring temperatures (Hinke et al., 2012; Lynch et al., 2012; Walther et al., 2002). Air temperature was an informative predictor of CIDs but it was also highly correlated to sea ice conditions rather than precipitation (Figure S2). Interestingly, October air temperature was not as highly correlated to November snow depth at Palmer Station and cannot explain the variation in snow depth recorded on the two islands. Past studies, including some that included the HUM data analyzed here, used October air temperature as a predictor of CIDs (Hinke et al., 2012; Lynch et al., 2009, 2012) with the assumption that temperature was an indicator of precipitation, but our results suggested this is not the case at Palmer Station. Air temperature could be indicative of snow characteristics (packed or slushy) that can influence nest site flooding but as is the case for a majority of landscape and population‐scale wildlife studies (Boelman et al., 2019), we lack the type of snow data that would be required to test this idea. We thus interpret the association between air temperature and CID as being related to sea ice conditions. For other WAP studies that found air temperature was related to CID, the main mechanism could be through sea ice and not precipitation (i.e., Hinke et al., 2012; Lynch et al., 2009; Lynch et al., 2012).

### 4.4 The importance of environmental variability in driving clutch initiation dates

Phenological variability in penguins can occur with or without environmental variability. While it has been suggested that high variability in penguin breeding phenology is normal under both stable (e.g., penguin colony at a zoo) and variable environments (e.g., penguin colony in nature, Youngflesh et al., 2018), the tight connection between phenology and the environment under variable conditions in our study emphasizes phenology can be a reliable indicator of environmental forcing. For example, at TOR ~ 80% of the variation in CIDs was explained by the environment, suggesting that 20% of the variation may have been due to other factors such as inaccuracies in weather data collection (e.g., snow was not measured on each island), parental age/experience/condition (Ainley & Schlatter, 1972; LeResche & Sladen, 1970; Moreno, Leon, Fargallo, & Moreno, 1998; Trivelpiece, Butler, Miller, & Peakall, 1984) or colony size/nest location (Barbosa, Moreno, Potti, & Merino, 1997; Fargallo et al., 2006). In contrast, at HUM where only ~ 60% of the variation in CIDs was explained by the environment, there could be more random individual variation because weather impacts were simply less severe. Therefore, synchronous breeding with lower variability in phenology is more likely to occur when a factor delays breeding, such as penguins waiting for snow to melt to lay eggs at TOR. In comparison, at HUM (or at a zoo), where conditions are often more predictably suitable and birds can begin breeding as they are ready, higher variability in phenology and less synchrony may occur. Notably, and supporting our rationale, a captive penguin population exhibited less synchrony among individuals each year than a wild population (Youngflesh et al., 2018). Penguin life histories are the product of evolution; hence, their selection must scale to environmental thresholds that optimize reproductive strategies (Fraser & Trivelpiece, 1996). Because Adélie penguin life history is tied overall to short seasonal cycles (Fraser & Trivelpiece, 1996), these environmental thresholds may function as critical drivers of breeding phenology that may result in more synchrony and less variability when the specific parameters associated with these thresholds are exceeded.

### 4.5 Effect of clutch initiation date on breeding success and the role of match‐mismatch dynamics

Penguin chick fledging mass and breeding success can be affected by numerous factors (see Hinke et al. (2012) for an overview), which can represent the investment of parents in their chicks and also be due to factors that are unrelated to CIDs (e.g., Youngflesh et al., 2017). We found no relationship between CID and chick fledging mass, which corroborates a past study at this location (Cimino et al., 2014). At both islands, breeding success was higher for earlier breeders but the relationship was not statistically significant at HUM, suggesting the ramifications of snowfall were less severe due to lower snow accumulations.

Similar to our study, on a circumpolar scale, Adélie penguin breeding success was higher when penguins bred earlier (Youngflesh et al., 2017) making it clear that either late CIDs contribute negatively to breeding success, or conditions that contribute to late CIDs also influence breeding success negatively. For example, snow accumulation that caused late CIDs will eventually melt and can then potentially decrease the survival of eggs or chicks through nest flooding. In this instance, synchronous breeding of a colony cannot outweigh the importance of synchronizing lay dates with optimal environmental conditions (in contrast to the hypotheses of Youngflesh et al., 2017), as nest flooding can lead to near‐immediate nest failure. Earlier breeding could also signify other optimal habitat conditions, such as adequate food availability, but Youngflesh et al. (2017) suggested that while mismatches in Adélie penguins are apparent, it is not the main driver of reproductive dynamics.

It may be important to also consider phenological mismatch in the context of the latitudinal climate gradient along the WAP, which is characterized by a warmer, maritime climate in the north that is nearly sea ice‐free most of the year and a colder, continental climate in the south where some perennial sea ice still exists (the transition zone is near Palmer Station, Ducklow et al., 2013). Along this gradient the timing of sea ice retreat can affect the timing and magnitude of phytoplankton blooms differently, as well as the potential for temporal mismatches in trophic interactions. For example, the northern WAP showed less favorable environmental conditions for phytoplankton blooms when the ~1970s were compared to the ~1990s, whereas the southern WAP experienced more favorable conditions (Montes‐Hugo et al., 2009). In the northern WAP, environmental changes not only led to decreased phytoplankton biomass, but also a cascade of other changes, including a shift to smaller phytoplankton species (Montes‐Hugo et al., 2009), smaller and less abundant microzooplanton (Garzio & Steinberg, 2013), lower lipid content krill (Ruck, Steinberg, & Canuel, 2014), shifts in macrozooplankton communities (Steinberg et al., 2015), decreased Adélie penguin populations (Lynch & LaRue, 2014) and a microbial (vs. krill) based food web (Sailley et al., 2013). Thus, despite large‐scale sea ice decreases in extent and duration along the entire WAP (Ducklow et al., 2013), the north and south regions started with different baselines, thus progressed differently along the Adélie optimal‐suboptimal curve (e.g., Fraser & Trivelpiece, 1996; Smith et al., 1999).

While Youngflesh et al. (2017) aimed to detect phenological mismatches between Adélie penguins and bloom onset/ice retreat, they did not include bloom magnitude, which has cascading effects on higher trophic levels. Further, it is unclear how these indices relate to macrozooplankton phenology, a factor that Steinberg et al. (2015) admittedly could not address. Therefore, for future studies investigating trophic mismatches, it is important to first consider the baseline from which change is occurring, because change can either be toward less or more favorable conditions (thus affecting trophic interactions differently). If long‐term warming persists along the WAP, then less favorable conditions will continue to shift southward, as will the increased potential for trophic mismatches.

Further, flexible reproductive timing may provide some buffer for reproductive success but it may be limited by both CID and snow. High breeding success was seen when CID was <day 45 and snow depth was <20 cm. In contrast, the two years (2001 and 2015) with the greatest snow depths had some of the lowest breeding success values and latest CIDs; this pattern was also noted at the Copacabana colony (~400 km north of Palmer Station, Hinke et al., 2012). During years of such environmental stress, life history strategies are in effect being tested by the environment, and the high frequency of nest failures highlights an incompatibility between Adélie penguin life history and precipitation above an optimal threshold.

### 4.6 Trends in clutch initiation dates placed into an environmental context

The WAP has been warming since at least the 1950s with increasing winter air temperature and precipitation (Kirchgäßner, 2011). During our study period, there was no trend in precipitation, and after decades of sea ice decline from 1970 to 2009 (most notably the ice season became shorter), sea ice began to rebuild along the WAP after reaching a local minimum in 2008–2009 (i.e., the annual ice season became longer post‐2008–2009) (Henley et al., 2019; Schofield et al., 2017). In general, to detect an ecologically relevant trend in phenological datasets it may be necessary to consider and evaluate environmental conditions over the same temporal scales. For example, no trend in Adélie penguin CIDs over our 25‐year time series should be expected given the absence of trends in environmental data. However, a trend was observed only after assessing the CID time series pre‐/post‐2009, which demarcates when there was a change in sea ice trends (as mentioned above). Modeled and observed CIDs trended toward earlier dates from 1979 to 2009 but reversed toward later dates as ice increased from 2010 to 2016. Similar negative trends in Adélie penguin phenology at Admiralty Bay and Palmer Station were observed from ~1990 to ~2000/~2010 including some years after the sea ice reversal (Lynch et al., 2012; Youngflesh et al., 2017); however, Youngflesh et al. (2017) deemed these trends biologically insignificant without putting the results into an environmental context.

For future studies, after identifying the correct environmental variables (which is inherently difficult in natural systems), we suggest that phenological data should be matched along the same time scales to long‐term climate data to determine whether a trend can be expected and detected. It is also important to recognize the impact of landscape geomorphology at a study site to understand how weather (e.g., wind and precipitation) may differentiate impacts on colonies located in close or distant proximity. This is especially relevant when looking at phenology across latitudes, where a delay in phenology at southerly latitudes could be amplified or reduced by landscape effects (e.g., Lynch et al., 2012). The influence of landscape geomorphology on nest site microclimate may also be an important factor in identifying Adélie penguin refugias under future climate change scenarios. Regardless of the mechanism driving phenological variability, reproductive success is generally higher for species able to adjust their breeding phenology and strategy to climate change (Forcada et al., 2008; Visser & Both, 2005) and for Adélie penguins, their responses to climate change may partly depend on this flexibility (Ballerini, Tavecchia, Pezzo, Jenouvrier, & Olmastroni, 2015). The ability of Adélie penguins to track environmental variability demonstrates phenotypic plasticity in their behavior and simultaneously emphasizes vulnerabilities in their life history to increased precipitation.

## CONFLICT OF INTEREST

The authors declare no competing interests.

## AUTHOR CONTRIBUTIONS

WRF designed the data collection methodology. WRF and DPF collected the data. MAC and WRF developed the research questions. MAC analyzed the data, and MAC wrote the initial manuscript with contributions from WRF. SS analyzed the sea ice data. All authors contributed to data interpretation, edited, and reviewed the manuscript.

## Data Availability

Data reported in this manuscript is either publically available or will be available online through the Palmer LTER Data System (http://pal.lternet.edu/data/) within one year of publication.
