# Supplementary material for: The interaction between island geomorphology and environmental parameters drives Adélie penguin breeding phenology on neighboring islands near Palmer Station, Antarctica
Source: Ecol Evol. 2019 Jul 31;9(16):9334–49. doi: 10.1002/ece3.5481 (PMC6706194; doi:10.1002/ece3.5481)
Supplement: Supplementary file 1 [file ECE3-9-9334-s001.pdf]

SUPPLEMENTAL INFORMATION

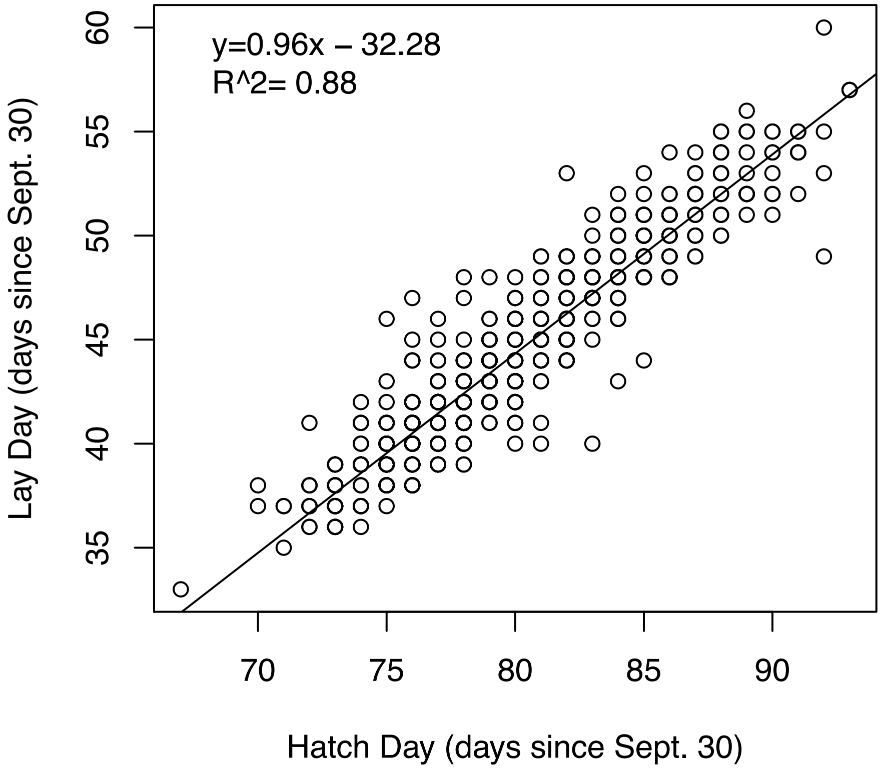

Supplemental Figure 1. Fitted linear relationship between true lay and hatch days for egg 1 on Humble and Torgersen Island.

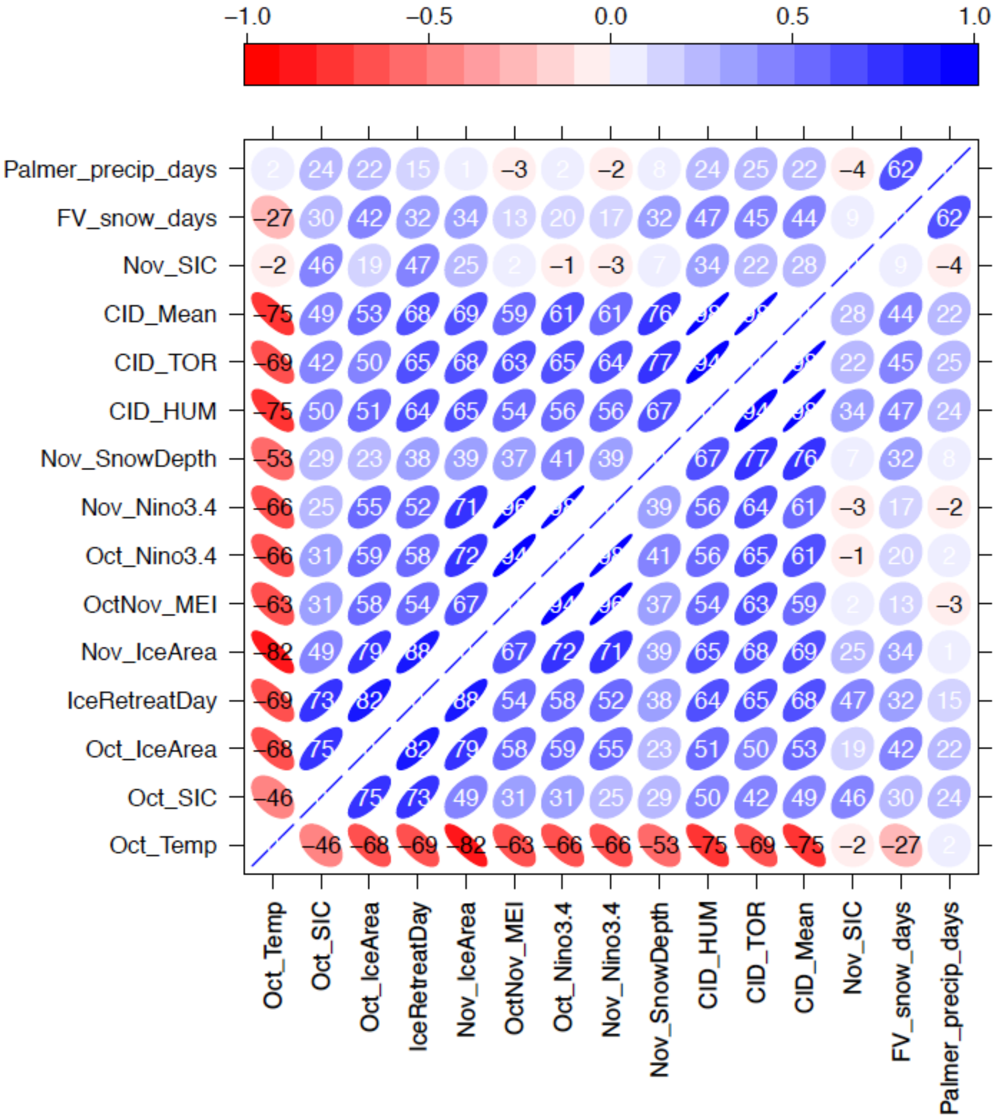

Supplemental Figure 2. Correlogram showing correlation coefficients using Pearson's correlation between clutch initiation date (CID) and ice and weather environmental predictors. The intensity of color shading reveals the correlation with warm colors indicating a negative correlation and cool colors indicating a positive relationship. Circles indicate no relationship and ellipses indicate a stronger relationship. For legibility, the values on the plot range from -100 to 100 but can be interpreted as Pearson's correlation ranging from -1 to 1. Variables include: Palmer snow and rain days in October (Palmer_precip_days), Faraday/Vernadsky snow days in

October (FV_snow_days), October air temperature (Oct_Temp), October/November sea ice concentration (Oct_SIC/Nov_SIC), October/November sea ice area (Oct_IceArea/Nov_IceArea), Sea ice retreat day (IceRetreatDay), October to November MEI (OctNov_MEI), October/November Nino 3.4 index (Oct_Nino3.4/Nov_Nino3.4), November snow depth (Nov_SnowDepth), ClD at Humble (CID_HUM), CID at Torgersen (CID_TOR) and mean CID at Humble and Torgersen (CID_Mean).

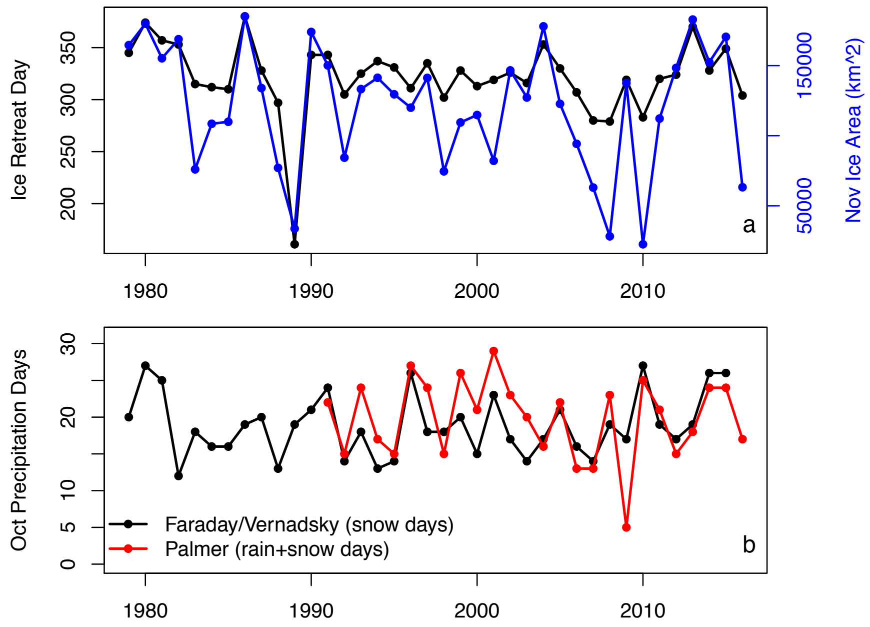

Supplemental Figure 3. Time series of sea ice and precipitation data from 1979 to 2016. **a)** Sea ice retreat day (Julian day, days since January 1) and November sea ice area over time. **b)** The number of precipitation days in October at Faraday/Vernadsky and Palmer Station. Precipitation records between the two locations displayed the same variability over time and were correlated (R=0.63; R = 0.71 without the 2009 outlier). The discrepancy in precipitation events in 2009 may be due to missing rain records at Palmer Station.
